# Commentary on: Offering No-Cost Cosmetic Revisions: The Experience of an Academic Cosmetic Surgery Program

**DOI:** 10.1093/asjof/ojad036

**Published:** 2023-04-11

**Authors:** Al Aly

See the Original Article here.

In this paper,^1^ the authors describe a “no cost cosmetic revision policy.” They found their revision rate was reasonable and helped their practice. They also surveyed a number of mostly private practices and found the majority did not offer a no-cost revision policy. To me, a no-cost policy is an “entitlement” with potential negative side effects. It is like the soft-drink refill policy in restaurants. If the refill is for free, I am more likely to get a refill than if I had to pay for each drink. Consciously or unconsciously, the customer/patient's expectations are adjusted based on the presenting policy. Also, in my experience, the success of a no-cost policy is going to depend on the surgeon's particular patient population (Video).

Lastly, I share an alternative policy: “The patient is responsible for anesthesia/facility fees, and a variable amount of the surgeon's fee (based on the practice setting).” Once a revision is deemed necessary, the surgeon can decide how much, if any, the patient is responsible for. Whatever fee reduction is given at this point, it will be thought of as a gift not an entitlement.

## Supplementary Material

ojad036_Supplementary_Data
